# Ion exchange: an advanced synthetic method for complex nanoparticles

**DOI:** 10.1186/s40580-019-0187-0

**Published:** 2019-06-03

**Authors:** Geonhee Cho, Yoonsu Park, Yun-Kun Hong, Don-Hyung Ha

**Affiliations:** School of Integrative Engineering, Chung-Ang University, 84 Heukseok-ro, Dongjak-gu, Seoul, 06974 Republic of Korea

**Keywords:** Nanocrystal synthesis, Chemical transformation, Shape control, Composition control, Phase control, Heterostructure

## Abstract

There have been tremendous efforts to develop new synthetic methods for creating novel nanoparticles (NPs) with enhanced and desired properties. Among the many synthetic approaches, NP synthesis through ion exchange is a versatile and powerful technique providing a new pathway to design complex structures as well as metastable NPs, which are not accessible by conventional syntheses. Herein, we introduce kinetic and thermodynamic factors controlling the ion exchange reactions in NPs to fully understand the fundamental mechanisms of the reactions. Additionally, many representative examples are summarized to find related advanced techniques and unique NPs constructed by ion exchange reactions. Cation exchange reactions mainly occur in chalcogenide compounds, while anion exchange reactions are mainly involved in halogen (e.g. perovskite) and metal-chalcogenide compounds. It is expected that NP syntheses through ion exchange reactions can be utilized to create new devices with the required properties by virtue of their versatility and ability to tune fine structures.

## Introduction

Unlike a bulk material system, the advantage of nanomaterials is the ability to control their physical and chemical properties depending on their size and shape. These unique properties provide great opportunities for a wide range of applications, such as electrocatalysts [1–7], photovoltaic cells [8–11], batteries [12–15], sensors [16–19], biomedical [20–22] and electronic devices [23–25]. Thus, many studies have been focused on methods to tune the physical size, phase, and nano-dimensional shapes such as nanospheres, nanorods (NRs), and nanoplates [26–33].

Among many chemical and physical methods, solution-based colloidal synthesis has been widely studied because the size and shape of the nanoparticles (NPs) can be readily adjusted and uniform NPs can be achieved [34–40]. This method is a bottom-up approach in which the NPs are formed on the atomic or molecular scale as building blocks. In general, colloidal synthesis is carried out at a high temperature using inorganic or organometallic precursors and ligands. The synthetic process follows the La Mer model in which the growth of NPs to a certain size occurs after the nucleation step [41]. Nucleation of colloidal-based NPs can be initiated by the hot-injection technique [42, 43], which comprises the rapid injection of precursors onto hot mixtures. The morphology and composition of NPs are controlled through a combination of the precursors, ligands, reaction temperatures, reaction times, etc. These protocols have been well established and various classes of material with narrow size distributions can be obtained [35, 38, 44–51]. However, the development of new synthesis strategies is still required since new functionality and enhanced properties of NPs are always in demand. As an alternative to the conventional colloidal synthesis method, the ion exchange of NPs through a secondary chemical transformation has recently been developed [52, 53]. The ion exchange reaction is initiated by providing new ions to a template NP compound. This technique has received considerable attention because it can overcome the limited structures and compositions fabricated by traditional synthesis methods. Most of the NPs formed by directly colloidal synthesis have the stable phase, composition, and morphology to satisfy the thermodynamic conditions [54]. Contrarily, the ion exchange synthesis can lead to NPs in a non-equilibrium state by the rapid replacement of ions by solid-state diffusion, even at room temperature [52, 55, 56]. This secondary transformation synthesis allows kinetically controlled NPs which are not accessible by traditional direct synthesis routes. Thus, the NPs transformed by an ion exchange method often yield heterostructures [57], core–shell structures [58–63], and metastable phases [55, 64] that are difficult to achieve via conventional syntheses. Numerous applications were reported by utilizing ion exchanged-NPs in various devices such as solar cells, photocatalysts, and lithium batteries. In such applications, their performances were enhanced when the NPs were synthesized via ion exchange method [59, 60, 65–68].

In ion exchange reactions, the ions of the parent NP compound diffuse out from the lattice and become solvated by solvents. At the same time, the substituted ions are introduced into the lattice through inward diffusion. Especially, NPs can adopt higher diffusion rates than those occurring in bulk syntheses because they have a large volume-to-surface area ratio. The ion diffusion process in the lattice structure of NPs can be influenced by kinetic factors such as the reaction zone [69], lattice framework [70], density of vacancies [71], and interstitial sites [70]. The reason why NPs in metastable phases and shapes can be formed is due to the unique kinetic mechanism of the ion exchange reaction. Additionally, a thermodynamic driving force can be introduced as a tool for predicting the spontaneity of ion exchange reactions such as lattice energy [72], the solubility of the ions [73, 74], and hardness [75, 76]. Ion exchange reactions can be represented by the complex action of kinetic and thermodynamic concepts. This review covers the mechanism of ion exchange reactions influenced by kinetic/thermodynamic factors and introduces examples of NP synthesis through ion exchange reactions.

## Cation exchange

Generally, high mobility of ions in a lattice is required for facilitating ion exchange by solid-state diffusion. The diffusion of cations can easily occur since their ionic radii are generally smaller than those of anions. Due to this main advantage, a cation exchange reaction is more likely to occur than an anion exchange one. The compositions of NPs can be efficiently tuned by cation exchange that maintains the original NP morphology and in some cases, changes it via structural reorganization. Cation exchange reactions often lead to new products with complex structures such as heterostructures and metastable phases that are not accessible through conventional synthetic methods.

We start this review by introducing the kinetic and thermodynamic factors to understand the mechanism of cation exchange reactions. Next, we summarize examples of cation exchange reactions in group I, II, and IV metal-chalcogenide compounds, which are the most studied cases among the cation exchange reactions in NPs. We also cover cation exchange reactions that occur within other transition metal compounds. In addition, we show the evolution in the characteristics of the NPs through cation reactions and their device applications.

### Kinetic factors

Cation exchange reactions proceed through the inward diffusion of new ions and the outward diffusion of host ions. The exchange of cations often occurs in a non-equilibrium state dominated by kinetics rather than thermodynamics. To fully understand the mechanism, it is important to identify the kinetic factors that influence ion diffusion. The focus in this section is cation exchange reactions by introducing the factors related to dynamics, such as the reaction zone, lattice structure, and defects in the crystal structure.

#### Reaction zone

The reaction zone is the region where substitution reactions by solid-state diffusion take place between the parent and product NPs [69]. This concept can be used to predict the morphology and phase of the NPs after the cation exchange reactions. The difference in the diffusion rates between two cations controls the overall reaction rate of the ion exchange reaction and determines whether the whole surface or only a specific zone of the NPs behaves as the reaction region. The reaction zone is the region where inward diffusion of host ion and outward diffusion of newly ion occur simultaneously and ion exchange reaction occurs. If this region is the whole surface of template NPs, inter-diffusion occurs at the fully interface of NPs and a core shell structure can be formed. In addition, the reaction zone directly affects the morphology and the crystal structure of the product NPs. For example, the CdSe NR template can preserve the NR shape or transform it into a spherical shape of Ag_2_Se controlled by the reaction zone during cation exchange reactions [52]. If the reaction zone is the entire surface of the CdSe NR template, the anion sublattice can be deformed at the intermediate step of whole cation exchange reaction. At the same time, inter-diffusion of ions and anion/cation rearrangements occur at a non-equilibrium state. Finally, when the equilibrium state is reached, the shape of product NPs transforms to sphere because it is thermodynamically preferred. On the other hand, if the specific part of the NP crystal structure is the reaction zone, the local lattice distortion of the template NPs occurs at a level that is sufficient to maintain the structure of anion sublattice. In this case, the shape of the NR template shape can be maintained during the conversion of the chemical composition. The reaction zone is one factor influencing the morphological changes during cation exchange reaction. Since the driving force for the shape change is very complicated, many other factors such as the crystal structure should be considered to fully understand the morphology evolution during cation exchange reaction in NPs.

#### Lattice structure

The stability of the anion sublattice structure determines the overall morphology of the NPs after the cation exchange reaction. A volume change in the lattice structure of the template NPs can be caused by a distinctly different size between the host ions and the newly introduced ones. Morphological changes of template NPs can be determined by the degree of strain tolerance that the lattice structure can relieve when the new ions are introduced. When the critical point is reached by the stress applied to the lattice structure due to replacement by the new ions, void formation or shape changes from spherical to rod may be induced to relieve the stress. In the case of CdX (X=S, Se, or Te) NP templates, the morphological transformation by volume change can be developed through cation exchanges by Pd^2+^ or Pt^2+^ ions [74]. As the fractional volume change (∆V/V) from 25 to 46% occurs, the shape of the parent NPs is transformed to fragmented NPs or else voids are formed.

**Fig. 1:**
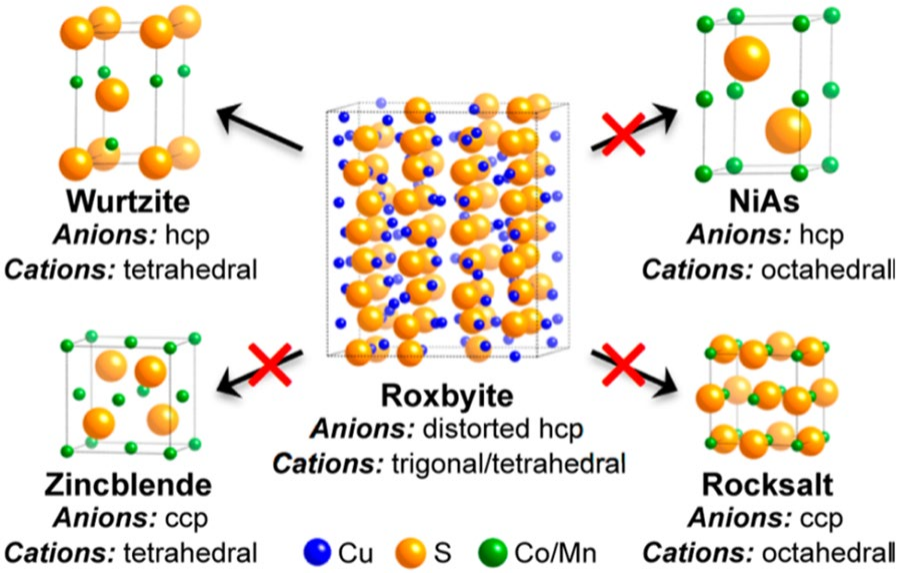
The crystalline structures of roxbyite Cu_2−x_S and wurtzite CoS/MnS. The selective transformation of template Cu_2−x_S NPs into CoS and MnS NPs is related to the conservation of the HCP anion lattice during the exchange of cations (Reprinted with permission from American Chemical Society [78])

The crystal structure of the product NPs can be determined by that of the template NPs. For example, the rock-salt structure of PbSe NRs controls the crystal structure of the final product, CdSe [77]; this can be zincblende or wurtzite, although the former is a less thermodynamically stable structure. Zincblende is closer in crystallographic symmetry with the template PbSe rock-salt structure, thus zincblende CdSe with a similar crystallographic structure to the PbSe template is formed by the cation exchange reaction. Similarly, when roxbyite Cu_2−x_S NPs are used as a template, wurtzite CoS and MnS can be obtained as products due to their comparable crystal structures [78]. Figure 1 shows the cation and anion framework of each crystal structure. The anion sublattice of roxbyite is a distorted hexagonal close-packed (HCP) structure, while the cation lattice structure is a mixed environment of trigonal and tetrahedral structures. The structure of the product NP is wurtzite, which is metastable in bulk, while the cation and anion frameworks are analogous to roxbyite. As shown, the crystallographic similarity between the template NPs and the products is an important measure in the ion exchange reaction to be able to predict the crystal structure of the product NPs.

**Fig. 2:**
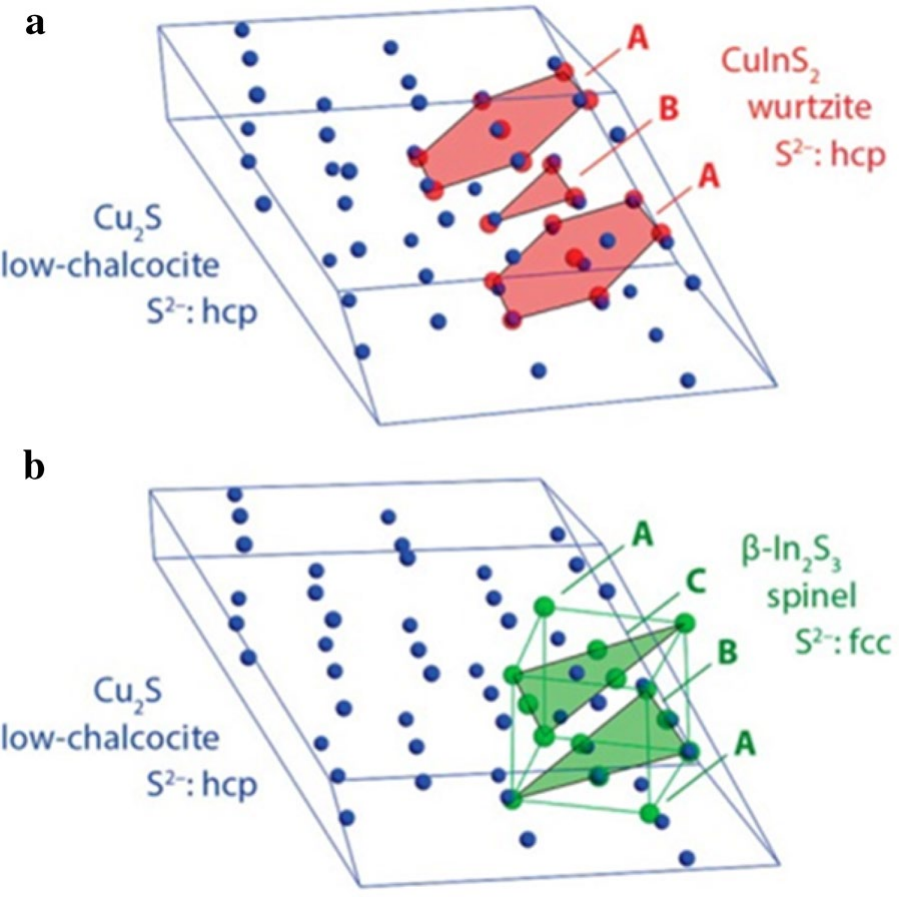
A comparison of the anion framework of template Cu_2_S NPs with CuInS_2_ and In_2_S_3_. **a** The anion sublattice of wurtzite CuInS_2_ has the same HCP structure as the template. **b** The anion sublattice of spinel In_2_S_3_ (FCC) is dislocated from the sublattice structure of the template (HCP) (Reprinted with permission from American Chemical Society [79])

Due to the difference in crystal structures between the template and the product, not only can the shape of the product be transformed, but also the degree of ion exchange can be limited. For example, two kinds of phase (CuInS_2_ and In_2_S_3_) can be obtained through the cation exchange of Cu_2−x_S with In^3+^ ions [79]. Figure 2a shows that the anion sublattice of Cu_2_S and the CuInS_2_ are similar and form an HCP atomic array in the product. On the other hand, Fig. 2b shows that the anion sublattice of In_2_S_3_ has a face-center cubic (FCC) structure and differs from the Cu_2_S HCP sublattice structure. For the preservation of the anion sublattice, the full exchange of the cations from Cu_2−x_S to In_2_S_3_ structure is restricted, and so CuInS_2_ is formed.

#### Defects

One of the most important factors for ion diffusion during a cation exchange reaction is defects such as vacancies and interstitial sites in a crystal structure [71, 77, 80–82]. When defects exist in the template NPs, the ion diffusion pathway having a low activation energy is formed, and the inter-diffusion of ions can be promoted along this pathway during cation exchange reaction. Groeneveld et al. [71] introduced the concept of a Frenkel pair for modeling a diffusion process in the presence of defects. When the cation exchange of Cd^2+^ into a ZnSe template occurs, a Frenkel pair acts to promote the external diffusion of Zn^2+^ ions. In this case, the activation energy required for the formation of the Frenkel pair can be overcome at high temperature so that the cation exchange reaction is spontaneously performed.

Vacancies in a crystal structure of template NPs can accelerate cation exchange reactions. For example, the ion exchange reaction of copper selenide is promoted by Cu vacancies [80]. When Cu^+^ is exchanged for Zn^2+^ and Cd^2+^, Cu_2−x_Se with a high vacancy density is more active than Cu_2_Se for the cation exchange reaction. Because the vacancy provides a pathway for ion diffusion, the activation energy for cation exchange is lower, which causes the more active cation exchange reaction from Cu_2−x_Se to ZnSe or CdSe than occurs in Cu_2_Se.

**Fig. 3:**
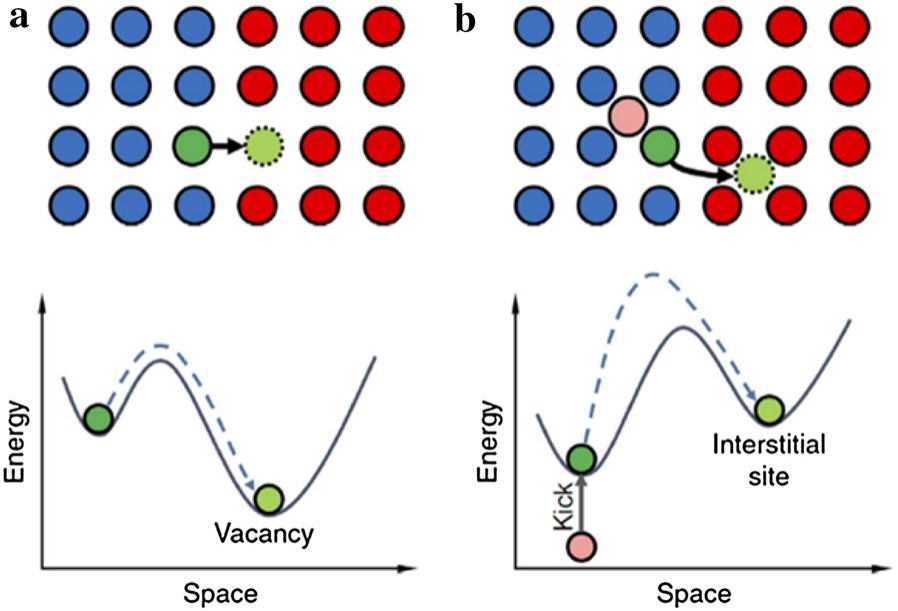
Illustration of the ion diffusion process via vacancies (**a**) and interstitial sites (**b**) at the PbS/CdS interfacial site. The blue and red circles represent Pd and Cd, and the pink circle is an interstitial site. These two processes have different activation energies due to the ‘vacancy-mediated’ and ‘kick-out’ mechanism. In this case, Cd ions diffuse through the interstitial site (**b**) is advantageous in terms of the activation energy (Reprinted with permission from Springer Nature Publishing AG [82])

Interstitial sites in the crystal structure of the template NP can also be an effective pathway for ion diffusion. Figure 3 shows the results of the activation energy analysis of two mechanisms through the cation exchange reaction between PdS and CdS [82]. Cd ions diffuse through the interstitial site (Fig. 3b) rather than the vacancy sites (Fig. 3a), which is advantageous in terms of lowering the activation energy. Likewise, the cation exchange process mediated by the vacancy and interstitial sites can be promoted because of the small activation energy required for ion diffusion.

### Thermodynamic factors

Not only can the spontaneity of the reaction be determined through the introduction of thermodynamic factors involved in cation exchange reactions, it also helps to select suitable ligands and solvents for each NP template.

#### The energy concept of cation exchange

It can be predicted whether spontaneous ion exchange reactions will occur due to the thermodynamic driving force [83]. Cation exchange reactions can be classified into four reaction steps [84], and the major thermodynamic parameters of each step are as follows [69, 74, 84]:

$${\text{C}} - {\text{X}}_{{({\text{crystal}})}} + {\text{M}}^{\text{n + }}_{{({\text{sol}}.)}} \to {\text{M}} - {\text{X}}_{{({\text{crystal}})}} {\text{ + C}}^{\text{n + }}_{{({\text{sol}}.)}}$$

1. C − X → C + X (dissociation)
2. M^n+^ → M (desolvation)
3. M + X → M − X (association)
4. C → C^n+^ (solvation)

In the process of dissociation and association, the lattice energies of the crystal structure influence the reaction process. The lattice energy of ionic crystals is the energy required to break bonds in the crystal structure at 0 K and separate it into individual ions. The larger the lattice energy, the more stable the crystal structure. A spontaneous exchange reaction can be predicted if the lattice energy of the product NP is larger than the parent NP.

The spontaneity of the reaction can be predicted in the desolvation and solvation steps through the solubility between the cation and the solvent [52, 73]. The solubility of the host cation in a solvent should be high for the ion exchange reaction to proceed well [75].

Since the volume-to-surface ratio of NPs is high, the surface energy affects solid-state exchange reactions [85]. However, it is not easy to calculate the surface energy because of the complexity of the ligand and/or surface lattice structure.

#### Parson’s hard and soft acids and bases (HSAB) theory

Colloidal NPs are usually surrounded by ligands which serve to stabilize the NP surface. Therefore, it is important to understand the interaction between the ligand and surface ions of NPs. The HSAB theory is used as a tool to predict the affinity among solvents, ligands, and ions. This theory infers that hard acids are preferred over hard bases and weak acids are adopted by weak bases [76]. If the host cation can form a more stable acid–base pair with a solvent/ligand than the pair of ingoing cation-solvent/ligand combination, the affinity between the host cation and the solvent/ligand is high. This high affinity can lead the cations to be removed from the template NPs. For example, when the soft acid cation (e.g. Cu^+^, Pb^2+^, Ag^+^) is the host cation and the hard acid cation (e.g. Zn^2+^, Cd^2+^, In^3+^) is the ingoing cation, the affinity between the host cation and soft base ligand is higher than the affinity between ingoing cation and ligand. Therefore, using soft base ligands (e.g. tri-*n*-octylphosphine) for soft acid cations in metal-chalcogenide NP templates would be thermodynamically favorable to facilitate an ion exchange reaction. Oleic acid and oleylamine (OA) are widely used as hard base ligands in cation exchange reactions forming stable metal ion-ligand complexes, which can support the solvation of the hard acid metal cation of the parent NP. In this case, hard acid cation (Zn^2+^, Cd^2+^, In^3+^) can be easily replaced by soft acid cations due to high affinity between hard base ligand and host cation. In addition, the affinity of the host cation and the solvent (e.g. ethanol, methanol, hexane) to promote the cation exchange reaction should be considered [73].

The HSAB theory suggests that a suitable solvent or ligand for a host cation will play a positive role in the exchange reaction. However, it is difficult to predict this exactly because the ligand and solvent are simultaneously involved in the ion exchange reaction and are influenced by many other variables, such as surface defects, decomposition of the ligand, and other chemical reactions. In addition, it is important to consider the kinetics since most ion exchange reactions occur in a non-equilibrium state.

## Cation exchanges in I–VI compounds ↔ II–VI compounds

Many studies have been progressed for the cation exchanges between I–VI compounds  and  II–VI compounds. The first mechanistic study of cation exchange in NP compounds is the transition from CdSe NPs to Ag_2_Se NPs demonstrated by Son et al. [52]. The morphology of the NPs when the cation exchange reaction from CdSe to Ag_2_Se occurs can be maintained or changed due to the reaction zone depending on the NP size. It has been confirmed that NP phases can easily be controlled in a short time through cation exchange because the kinetics of the reaction in NPs are much faster than in bulk reactions. Ag_2_Se NPs react with Cd^2+^ to transform back to CdSe, thus showing complete reversibility between CdSe and Ag_2_Se through cation exchange. Since this initial study, others on cation exchange reactions in group I and II chalcogenide NPs have been actively progressed to precisely control their morphology and/or phase.

**Fig. 4:**
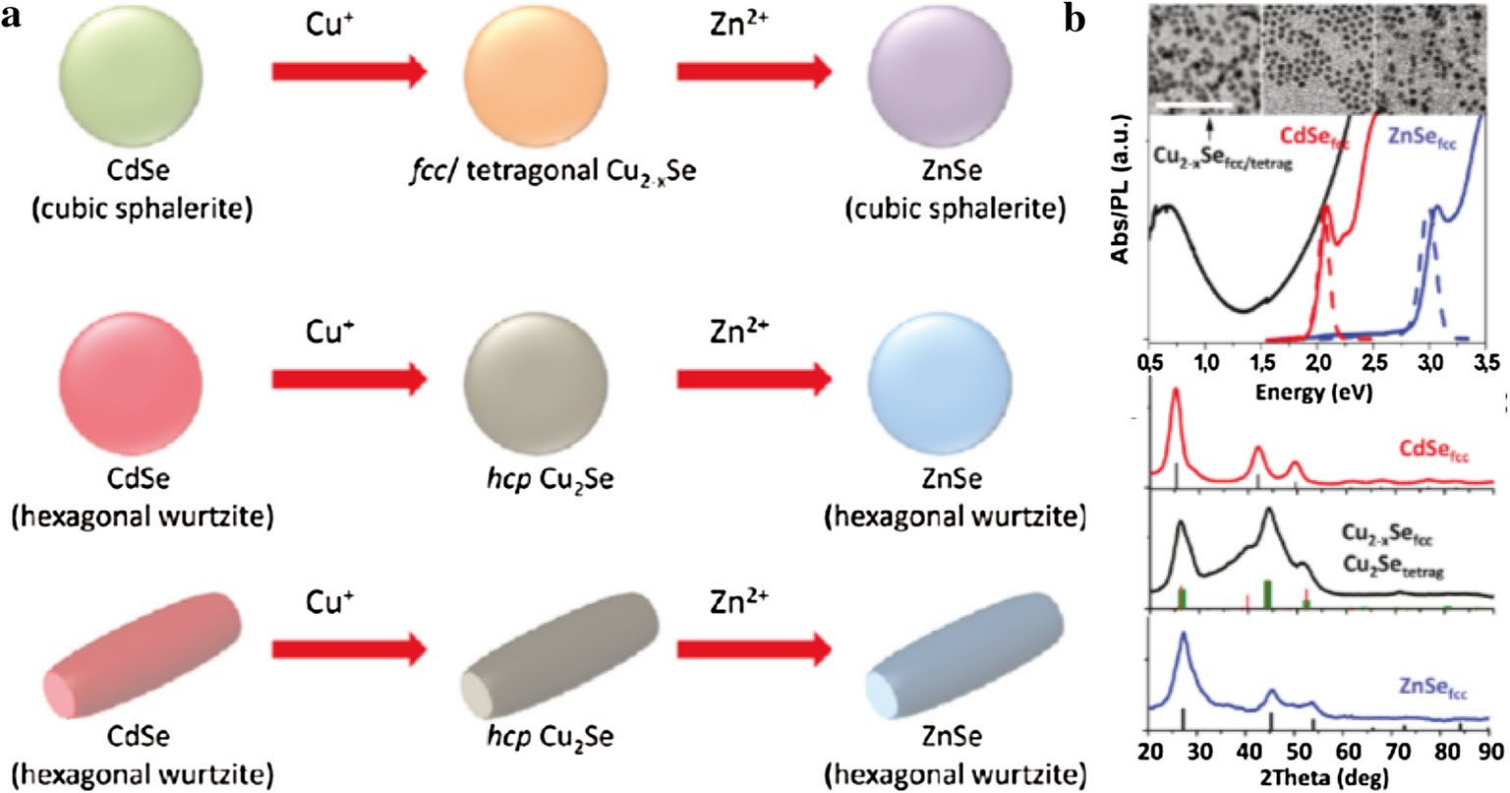
**a** A schematic of the cation exchange reaction initiating from CdSe NPs with various sizes, shapes, and crystalline phases. **b** A transmission electron microscopy image, optical absorption/emission spectra, and an X-ray diffraction pattern of cubic CdSe (black), FCC/tetragonal Cu_2−x_Se (red), and cubic ZnSe (blue) spherical NPs (Reprinted with permission from American Chemical Society [55])

One of the advantages of NP synthesis via cation exchange is the creation of NPs with a metastable phase, which is challenging by conventional synthesis methods. Li et al. [55] demonstrated sequential cation exchange reactions that the parent CdSe NPs was transformed to Cu_2_Se and ZnSe through cation exchange (Fig. 4). They confirmed that the crystal structures of Cu_2_Se and ZnSe are determined by the structures of the starting material, CdSe; although its crystal structure is changed, the morphology of the NPs was preserved when the cation exchange reaction occurred. When the CdSe with an HCP crystal structure is utilized, the intermediate Cu_2_Se and final ZnSe NPs have HCP crystal structure. Since the thermodynamically stable crystal structure of Cu_2_Se is FCC, the intermediate Cu_2_Se NPs with an HCP crystal structure are in a metastable phase whereas when CdSe with an FCC crystal structure is utilized, intermediate Cu_2_Se and final ZnSe NPs have FCC/tetragonal and FCC structures, respectively. The optical absorption/emission spectra of those NPs were changed when CdSe NPs were cation-exchanged to give Cu_2_Se and ZnSe, which provides a new pathway for the fine-tuning of the materials’ properties.

Cation exchange reactions also have the advantage of precise control of the NP phase. Zhang et al. [58] investigated the cation exchange reaction between CdS and Cu_2−x_S in NRs. Cu_2−x_S has several phases depending on the ratio of Cu to S (yarrowite: Cu_1.12_S, spionkopite: Cu_1.39_S, geerite: Cu_1.6_S, anilite: Cu_1.75_S, digenite: Cu_1.8_S, roxbyite: Cu_1.81_S, djurleite: Cu_1.96_S, and chalcocite: Cu_2_S). Since each Cu_2−x_S phase has different characteristics, it is necessary to fine-tune the phase to obtain the desired properties. When the cation exchange reaction occurs from CdS to Cu_2−x_S, the reaction proceeds in three steps: (1) Cu_2−x_S island formation, (2) core–shell CdS@Cu_2−x_S heterostructure formation, and (3) complete conversion to Cu_2−x_S. The phase of the resulting Cu_2−x_S NRs can be controlled by the reaction time and the amount of the copper precursor. When the reaction time is short and the amount of precursor is small, the Cu_2−x_S phase is roxbyite. However, when the reaction time and quantity of precursor are increased, the Cu_2−x_S phase exhibits low chalcocite after the djurleite phase has formed.

**Fig. 5:**
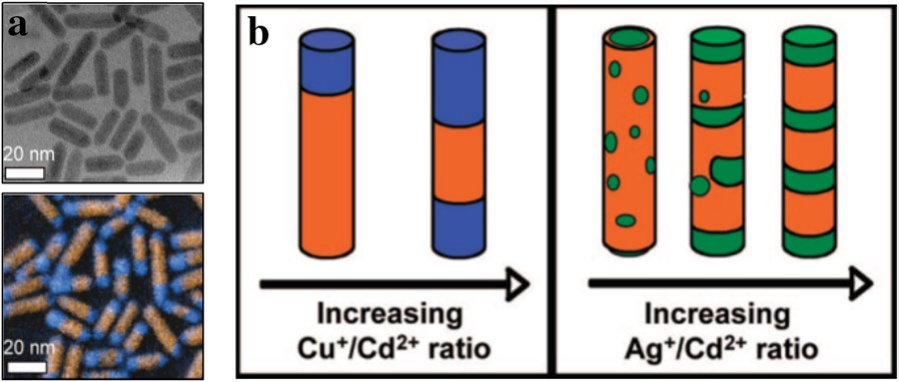
**a** A bright-field transmission electron microscopy image (top) and a color-composite energy-filtered transmission electron microscopy image (bottom) of CdS–Cu_2_S binary NRs The orange regions correspond to the Cd energy-filtered mapping and the blue regions correspond to the Cu-mapping. **b** A schematic of the structural transformation of the CdS–Ag_2_S and CdS–CuS NRs (Reprinted with permission from American Chemical Society [87])

**Fig. 6:**
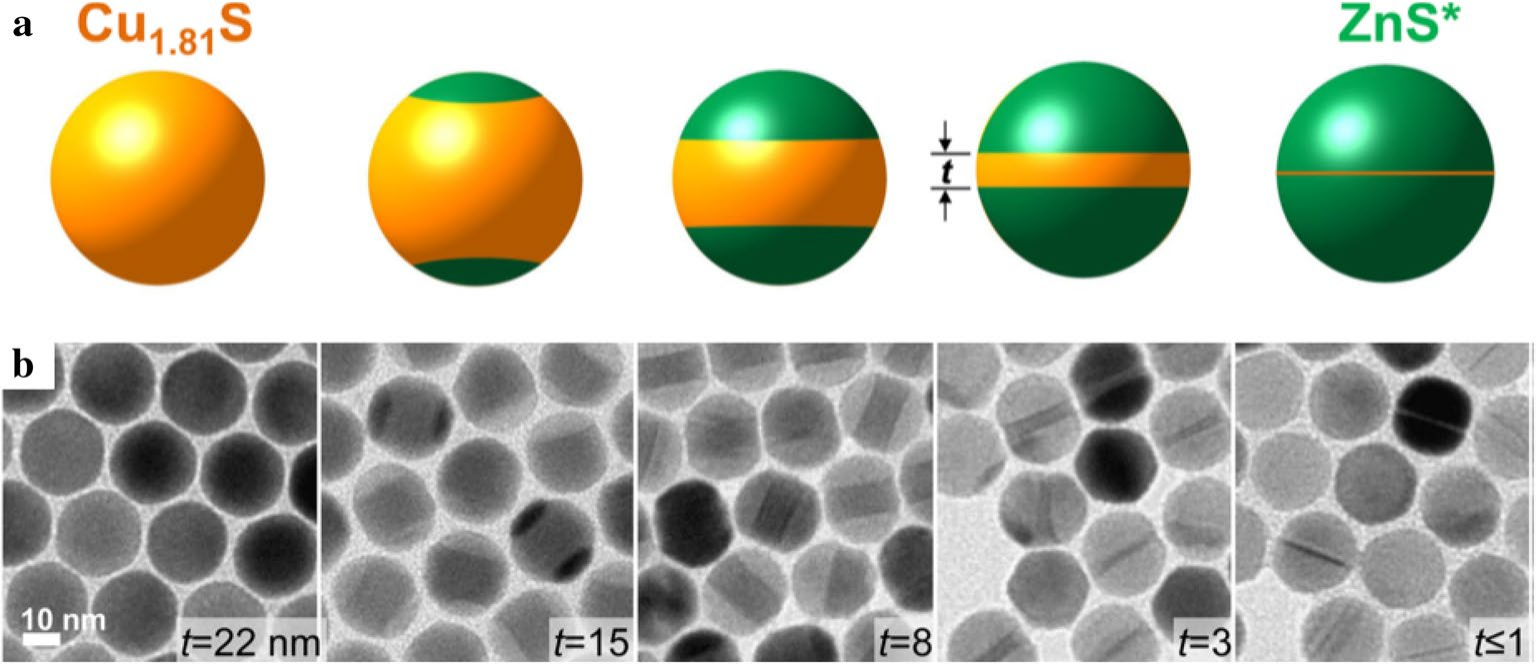
**a** An illustration of the cation exchange reaction of Cu_1.81_S into dual interface heterostructure particles with ZnS. **b** Transmission electron microscopy images showing the morphological change from the initial Cu_1.81_S NPs into ZnS with a Cu_1.81_S thin disk (Reprinted with permission from American Chemical Society [88])

The synthesis of NPs via cation exchange reactions provides a new pathway for designing complex-structured NPs such as core–shell or segmented NRs, which are difficult to obtain by conventional synthesis methods. Robinson et al. and Sadtler et al. synthesized CdS–Ag_2_S and CdS–Cu_2_S NRs through the partial cation exchange of CdS NRs and observed that the structure of the NRs changed depending on the choice of cation (Fig. 5) [86, 87]. When Cd^2+^ was exchanged by Ag^+^ ions, Ag_2_S segments with regular spacing induced by strain were formed in the NRs, resulting in superlattice CdS–Ag_2_S NRs. Unlike the cation exchange with Ag^+^, when the Cd^2+^ was exchanged with Cu^+^, binary heterostructured CdS–Cu_2_S NRs were created. This morphological difference was caused by the distinct chemical favorability and elastic distortion. Ha et al. [88] demonstrated the cation exchange from Cu_1.81_S to ZnS (Fig. 6). When the cation exchange from Cu_1.81_S to ZnS occurred, the reaction was initiated at both ends of the NPs, thereby forming a dual interface between Cu_1.81_S and ZnS. As the cation exchange reaction proceeded, the thickness of the Cu_1.81_S was reduced and the overall morphology of the NPs was transformed into nano-hamburger shapes. During the cation exchange reaction, not only did the Cu_1.81_S layer become thin, but also the phase of the copper sulfide part was converted from roxbyite to thermodynamically stable djurleite/low chalcocite and then changed back to roxbyite because of the strain. Moreover, localized surface plasmon resonance peaks appearing due to the Cu_1.81_S layer were tuned by controlling the thickness of the layer.

**Fig. 7:**
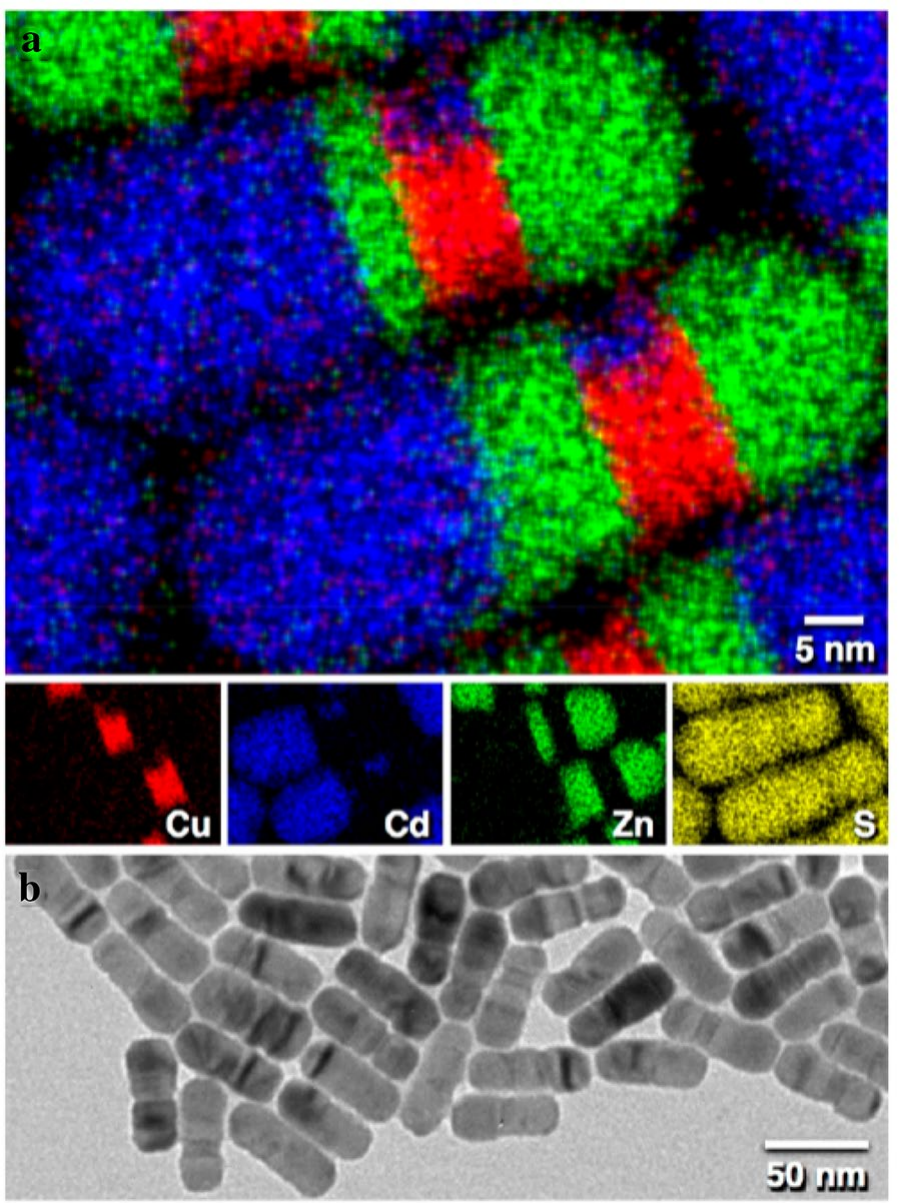
**a** Scanning transmission electron microscopy-energy dispersive X-ray spectroscopy image and **b** transmission electron microscopy image of CdS–ZnS–Cu_1.8_S–ZnS nanorod obtained from sequential cation exchange from Cu_1.8_S nanorod (Reprinted with permission from American Chemical Society [90])

Cation exchange is not limited to using only one cation; more than two kinds of cation can be used to create a more complex structure. Fenton et al. [89, 90] synthesized various NPs from G-1 (first-generation) nanostructures to G-3 nanostructures using cation exchange reactions with Cu_1.8_S. The Cu_1.8_S NPs were transformed to CdS and ZnS by cation exchange, and a structural change was observed depending on the reaction time. Other transition metals: Co, Mn, and Ni, as well as group II elements such as Zn and Cd, have been used for cation exchange to synthesize heterostructured NPs. Figure 7 shows CdS–ZnS–Cu_1.8_S–ZnS NPs which are fabricated by sequential cation exchange processes. It has been confirmed that multicomponent NPs can be synthesized by substituting the desired materials at the correct positions through a cation exchange method.

The NPs synthesized by cation exchange can be applied to various devices with enhanced properties and performance. Tang et al. [59] investigated a cation exchange reaction to synthesize CdS@Cu_2_S core–shell NRs from CdS NRs, which exhibited high performance as an absorbing layer in a solar cell. The device showed 5.4% energy conversion efficiency, which is higher than other NRs of similar materials that have been studied previously. Feng et al. [60] demonstrated the cation exchange reaction of a shell to ZnS in Au@AgAuS core–shell NPs, revealing a yolk-shell hybrid structure. These uniquely structured Au@ZnS NPs showed high photocatalytic activity due to optimization of the plasmon-exciton coupling and plasmon-enhanced electron–hole separation. Huang et al. [65] synthesized ZnSe NPs having a hollow structure by cation exchange and etching from Cu_2−x_Se NPs. The ZnSe NPs showed high photocatalytic activity because they have a hollow structure with a proper bandgap. Dogan et al. [66] demonstrated the formation of heterojunctions in NRs using the cation exchange from CdSe to Cu_2_Se. In a single nanowire CdSe, CdSe-Cu_2_Se heterojunctions were formed by a masked cation exchange reaction through electron-beam radiation. The masked cation-exchanged NRs can be utilized on-chip and to tune the properties of a device, and so it is expected that they will become useful in this respect.

## Cation exchanges in II–VI compounds ↔ IV–VI compounds

**Fig. 8:**
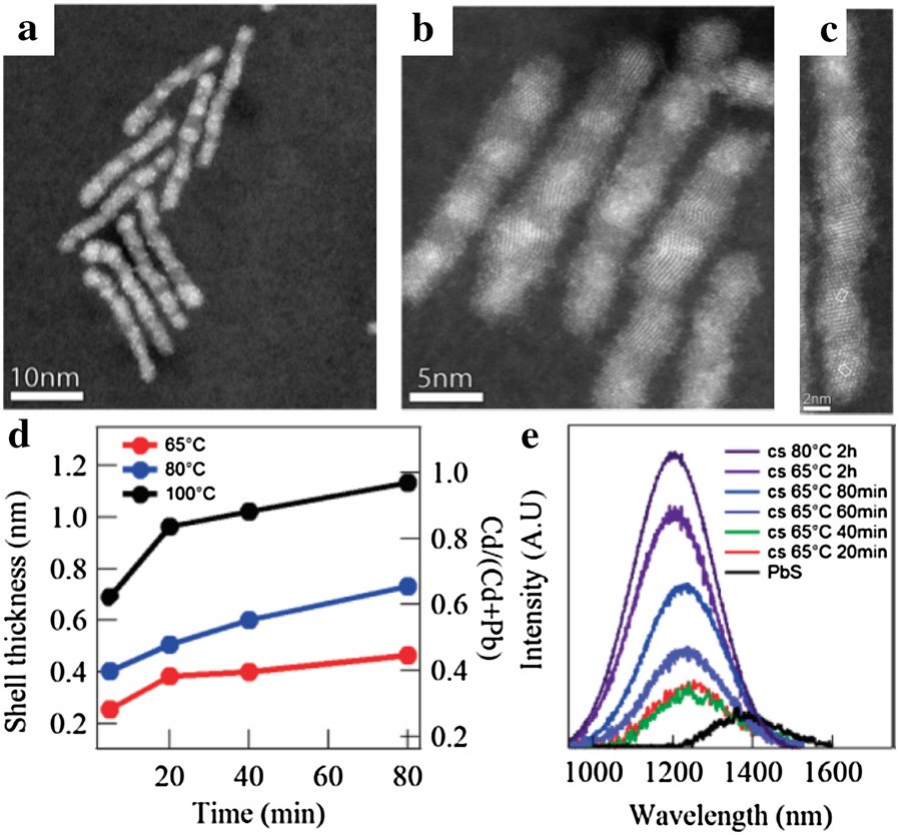
**a**–**c** Scanning transmission electron microscope-high angle annular dark field images of PbS/CdS rods showing multiple PbS dots inside the rods. **d** Ratio Cd/(Cd + Pb) and CdS shell thickness as a function of reaction time for different reaction temperatures. **e** Photoluminescence spectra with different reaction times for reactions at 65 °C and 80 °C (Reprinted with permission from American Chemical Society [91])

**Fig. 9:**
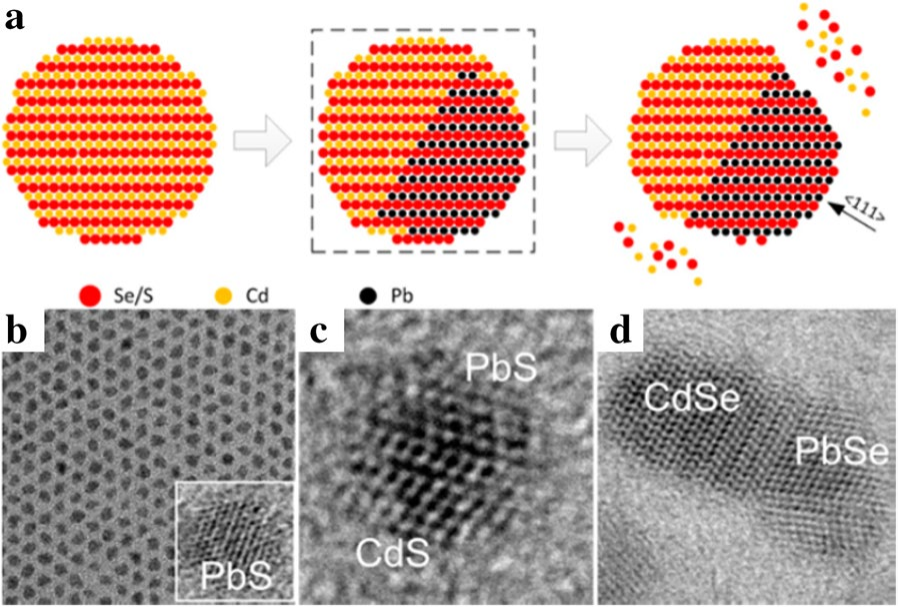
**a** A schematic of the cation exchange reaction in CdS or CdSe with Pb^2+^. Transmission electron microscopy images of PbS NPs synthesized from CdS NPs (**b**) and partially exchanged CdS NPs (**c**). Both their spherical nature and heterostructure can be observed. **d** A transmission electron microscopy image of partially exchanged CdSe NPs. The PbSe and CdSe domains form two (111) interfaces. Reprinted with permission from American Chemical Society [57]

Unlike the cation exchange reactions between I–VI and II–VI compounds, in which various elements have been used for cation substitution, the exchange reaction from Cd to Pb has been mainly studied as an example between II–VI and IV–VI compounds. Although PbX NPs generally show efficient emission in the infrared region, they have poor stability. Nevertheless, Pietryga et al. [61] demonstrated that cation-exchanged NPs can resolve this poor stability issue. PbSe/CdSe/ZnS core–shell–shell structured NPs were produced by a cation exchange reaction from PbSe NPs; they exhibited high stability against fading and a spectral shift due to optimized band alignment. Justo et al. [91] also designed complex-structured NPs, dot-in-rod PbS/CdS NRs, by partial cation exchange from PbS to CdS (Fig. 8). The photoluminescence (PL) spectra of these NRs were controlled by the cation exchange conditions such as reaction time and temperature, leading to the maximum PL quantum yield of 55%. The number of PbS dots in an NR was determined by the length of the starting material, CdS NRs. Zhang et al. [57] investigated to synthesize CdS–PbS Janus-like structured NPs through a controllable cation exchange reaction in CdS NPs (Fig. 9). The cation exchange reaction was developed along the <111> direction, thereby creating the Janus-like structured NPs that can be used to increase the efficiency of solar cells due to adjustable PL characteristics through cation exchange reaction parameters such as reaction time and temperature. Likewise, Yao et al. [92] investigated cation-exchanged NPs from CdS to PbS and their application in solar cells. When PbS NPs prepared by a cation exchange reaction were used in the photoactive layer of a solar cell, its efficiency was 7.89% and the performance was maintained for 110 days. In addition, Zhang et al. [67] synthesized PbSe_1−x_S_x_ NPs through cation exchange of CdSe_1−x_S_x_ to utilize the unique characteristics of PbSe_1−x_S_x_, which are different from pure PbSe or PbS. They confirmed that the morphology of the PbSe_1−x_S_x_ NPs was retained even when the ratio of Se to S was changed. The PL characteristics of the NPs were modified by cation exchange reaction time and S/Se stoichiometry.

## Cation exchanges in I–VI compounds ↔ IV–VI compounds

**Fig. 10:**
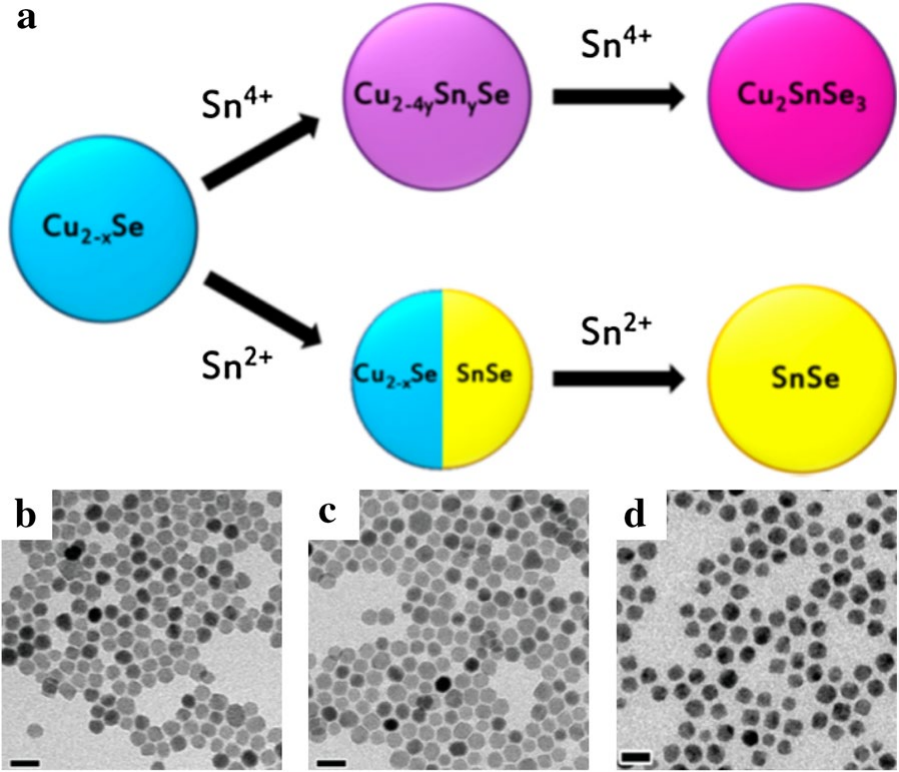
**a** A schematic of the cation exchange reaction in Cu_2−x_Se NPs involving Sn^4+^ and Sn^3+^ ions. Transmission electron microscopy images of pristine Cu_2−x_Se (**b**), Cu_0.66_Sn_0.33_Se (**c**), and SnSe (**d**) NPs (Reprinted with permission from American Chemical Society [93])

Cation exchanges between group I and IV chalcogenides mainly involve Sn. Unlike previous cations, Sn cations have several oxidation states, thus showing diversity in the morphology or phase of the synthesized NPs. De Trizio et al. [93] investigated the synthesis of Cu_2_SnSe_3_ or SnS NPs from Cu_2−x_Se by using two Sn cations with different oxidation states (Fig. 10). When Sn^2+^ and Sn^4+^ were used for the cation exchange reaction, the morphology of the NPs was maintained but the intermediate and final products were distinct depending on the oxidation state of Sn. When large-sized Sn^2+^ ions were used for the cation exchange reaction, orthorhombic SnSe NPs were formed through the intermediate Cu_2−x_Se/SnSe. On the other hand, Cu_2-4y_Sn_y_Se (y < 0.33) NPs were observed when small-sized Sn^4+^ was employed.

**Fig. 11:**
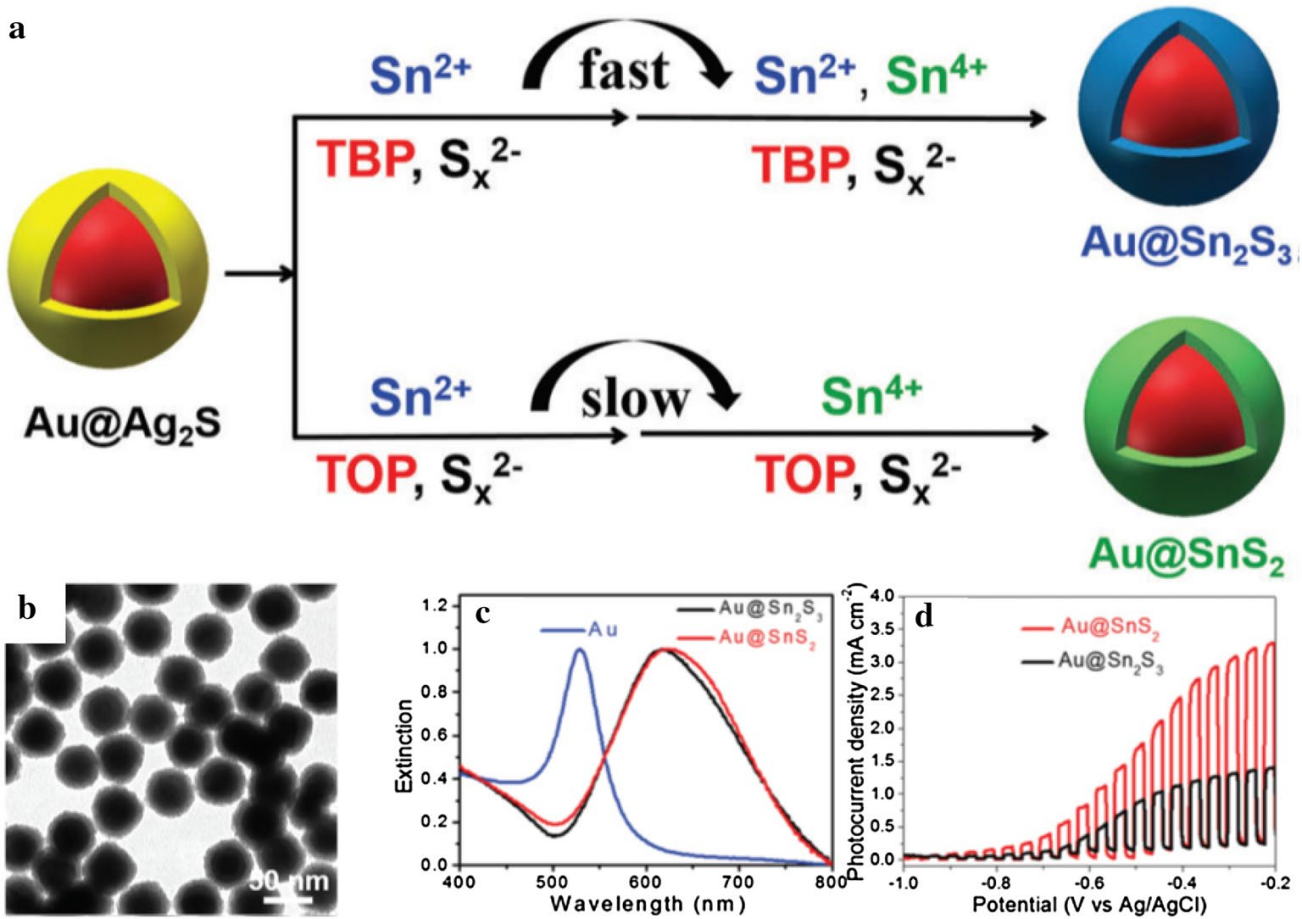
**a** The proposed mechanism for the formation of Au@SnS_3_ and Au@SnS_2_ core–shell NPs by using TBP or TOP as the ligand in the cation exchange reaction, **b** a transmission electron microscopy image of the resultant core–shell NPs using TBP, and **c** extinction spectra and **d** photocurrent-potential curves of the Au@SnS_3_ and Au@SnS_2_ core–shell NPs (Reprinted with permission from the Royal Society of Chemistry [63])

In recent years, Au@Sn_2_S_3_ or Au@SnS_2_ through cation exchange from Au@Ag_2_S core–shell NPs were synthesized by Cheng et al. [63] (Fig. 11). Both Sn^2+^ and Sn^4+^ were adopted for the cation exchange reaction and the final product influenced by the use of TOP (tri-*n*-octylphosphine) or TBP (tri-*n*-butylphosphine) as ligands. When TBP was used as the ligand, the cation exchange reaction proceeded rapidly and Au@SnS_3_ was synthesized as the final product. However, when TOP was utilized, the cation exchange occurred slowly and Au@SnS_2_ was created as the final product. These Au@SnS_2_ NPs exhibited high activity at − 0.2 V vs Ag/AgCl of 3.3 mA/cm^2^ when applied as a photoanode in a water-splitting device.

## Other cation exchange reactions

**Fig. 12:**
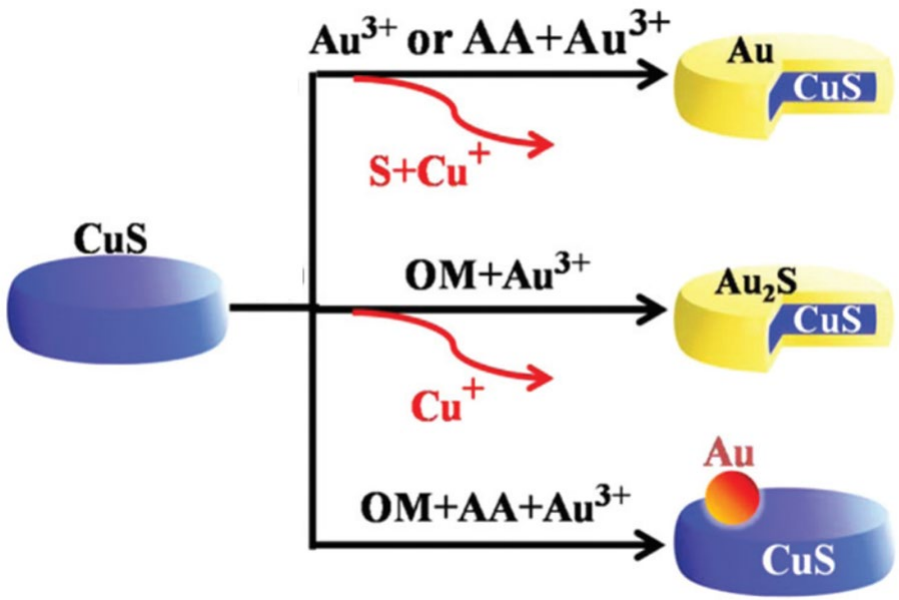
An illustration of the various reaction conditions and related heterostructures of Au or Au_2_S–CuS at room temperature (Reprinted with permission from the Royal Society of Chemistry [94])

In addition to the cation exchange reactions between the group I, II, and IV compounds as discussed earlier, many studies on those within the same group (i.e. I–VI compounds ↔ I–VI compounds) have been actively progressed. Wang et al. [62] demonstrated a cation exchange reaction to synthesize Au_2_S from Cu_2−x_S NPs. In this process, intermediate NPs were Cu_2−x_S@Au_2_S NPs having a core–shell structure, which is metastable in bulk. When the metastable Cu_2−x_S@Au_2_S core–shell NPs were irradiated with an electron beam, the NPs were converted into a dumbbell-like shape because the electron beam provided energy to transform them into a stable structure. The cation exchange reaction between Cu and Au in CuS was also observed by Hu et al. [94]. Through the cation exchange reaction from covellite CuS, various Au–CuS NPs with heterostructures such as CuS@Au core–shell NPs, CuS@Au_2_S core–shell NPs, and an Au/CuS dimer were synthesized by using ligands such as OA and ascorbic acid (Fig. 12). The near-infrared (NIR) absorption property of the cation-exchanged Au–CuS NPs was changed depending on their shape. Guo et al. [95] synthesized porous single-crystalline CdSe nanobelts by the cation exchange reaction of Cd^2+^ with ZnSe nanobelts. The synthesized CdSe nanobelts exhibited rapid, stable, and repeatable photoelectric properties when used as photodetectors.

**Fig. 13:**
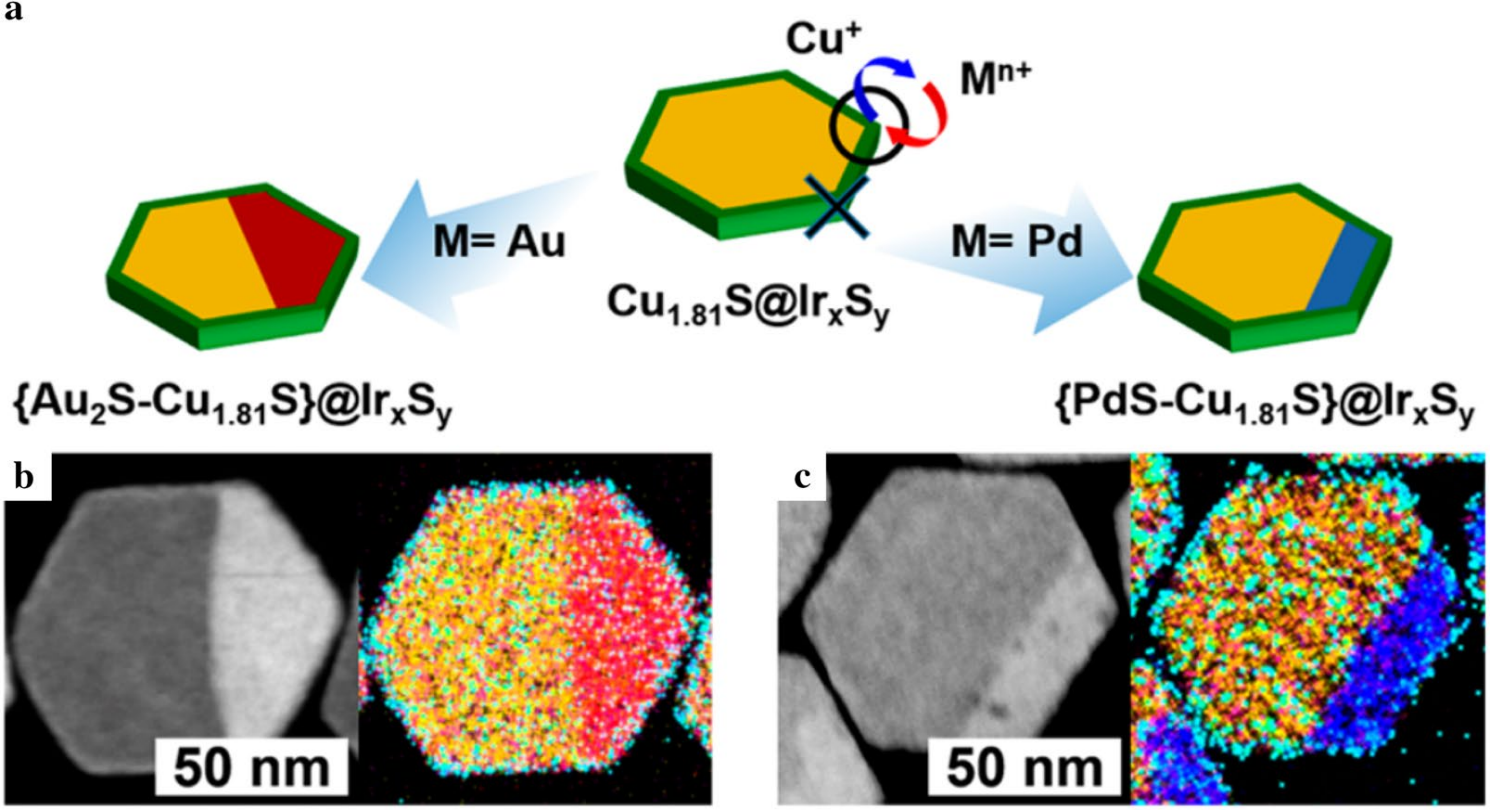
**a** A schematic of the cation exchange reaction in Cu_1.81_S@Ir_x_S_y_ involving Au and Pd ions. Scanning transmission electron microscopy images of the Janus-like (Au_2_S–Cu_1.81_S)@Ir_x_S_y_ NPs (**b**) and the Janus-like (PdS–Cu_1.81_S)@Ir_x_S_y_ NPs (**c**) (Reprinted with permission from American Chemical Society [96])

Furthermore, cation exchange reactions using other transition metals have also been widely studied. Fenton et al. [64] investigated the cation exchange reactions with Mn and Co in Cu_2−x_S. MnS and CoS NPs were yielded based on roxbyite and digenite Cu_2−x_S. When the roxbyite Cu_2−x_S was used as a template for cation exchange, wurtzite-structured MnS and CoS were synthesized. On the other hand, when the digenite Cu_2−x_S was utilized, metastable crystalline structured zincblende MnS and CoS were formed. Park et al. [96] synthesized (Au_2_S–Cu_1.81_S)@Ir_x_S_y_ nanoplates and (PdS–Cu_1.81_S)@Ir_x_S_y_ nanoplates with hexagonal and Janus-like structures by the cation exchange of Au and Pd in Cu_1.81_S@Ir_x_S_y_ (Fig. 13). When the cation exchange reaction occurred, the six corners of the hexagonal Cu_1.81_S nanoplates acted as cation exchange sites and the direction of the cation exchange was determined by the cation species. Based on this study, Pd_13_Cu_3_S_7_ NPs have been synthesized by the cation exchange reaction of Cu_1.81_S NPs [68]. Similar to the previous experiment, anisotropic diffusion appeared during the cation exchange leading to a Janus-like heterostructure. These Pd_13_Cu_3_S_7_ NPs showed high hydrogen evolution reaction catalytic activity requiring an overpotential of 64 mV vs. a reversible hydrogen electrode to achieve 10 mA/cm^2^.

Studies on cation exchange reactions have progressed not only in chalcogenide compounds but also in pnictide compound. De Trizio et al. [97] demonstrated the cation exchange reaction from hexagonal Cu_3−x_P to wurtzite InP. The copper vacancy in Cu_3−x_P promoted the cation exchange reaction to InP and the optical absorption property changed as the cation exchange reaction proceeded.

## Insight into anion exchange

Anion exchange is another case of conversion chemistry studied that has developed into a sub-area of ion exchange. Generally, anion exchange reactions are slower than cation exchange due to the low mobility and large ionic radii of anions [98]. Therefore, sluggish anion exchange often requires a longer reaction time and higher reaction temperature than for cation exchange. The benefits of the sluggishness can be utilized in partial anion exchange by controlling the slow reaction kinetics [99]. Cations can easily spread in template NPs while the anion sublattice of NPs is preserved due to the smaller ionic radii of the cations in cation exchange [98, 99]. Thus, cation exchange is usually arranged by an anion sublattice and the morphology of the NPs is not transformed. However, cation diffusion in anion exchange is often advanced and the cation sublattice is disrupted. Consequently, the morphology of NPs synthesized by anion exchange is transformed to hollow structures because of the ‘Kirkendall effect’ in most cases of the anion exchange of, for instance, metal-chalcogenide NPs [98].

The mechanism of anion exchange can be explained by the theories of mass action and thermodynamic energy. First, in anion exchange reactions explained by the theory of mass action [98], increasing the concentration of reagents in a solution can promote the kinetics to become similar to cation exchange. For example, metal sulfides have more positive Gibbs free energy of formation than metal oxides, but the anion concentration can induce an energy imbalance and facilitate the sulfidation reaction of metal oxides. However, the theory of mass action is not always applicable to the actual anion exchange reactions in NPs since there are many other factors controlling the overall reaction [100–102]. Second, the thermodynamic theory established for anion exchange is analogous to cation exchange [98]. The Gibbs free energy for a cation exchange reaction is affected by that of the compounds and the reduction potential of the cations. However, it is difficult to adapt the thermodynamic theory of cation exchange reactions to anion exchange because the anion source often requires decomposing or activating processes to become an active reagent. The emitted anions after the reaction can also cause uncontrolled additional reactions, such as O^2−^ ions into OH^–^. Thus, the thermodynamic spontaneity of anion exchange is determined by the precursors of the incoming anions and further reactions of the outgoing ions [98].

Research on anion exchange has been widely conducted on perovskites and metal oxides [99, 103–115]. The reaction temperature and time are generally different between the anion exchange reactions using halides with perovskites and the reactions of chalcogen groups with metal oxides. When anion exchange is performed in perovskites synthesized with halogen groups, the reaction requires much lower energy than for reactions from metal oxides to metal chalcogenides (a lower reaction temperature and a shorter reaction time) [98, 104].

**Fig. 14:**
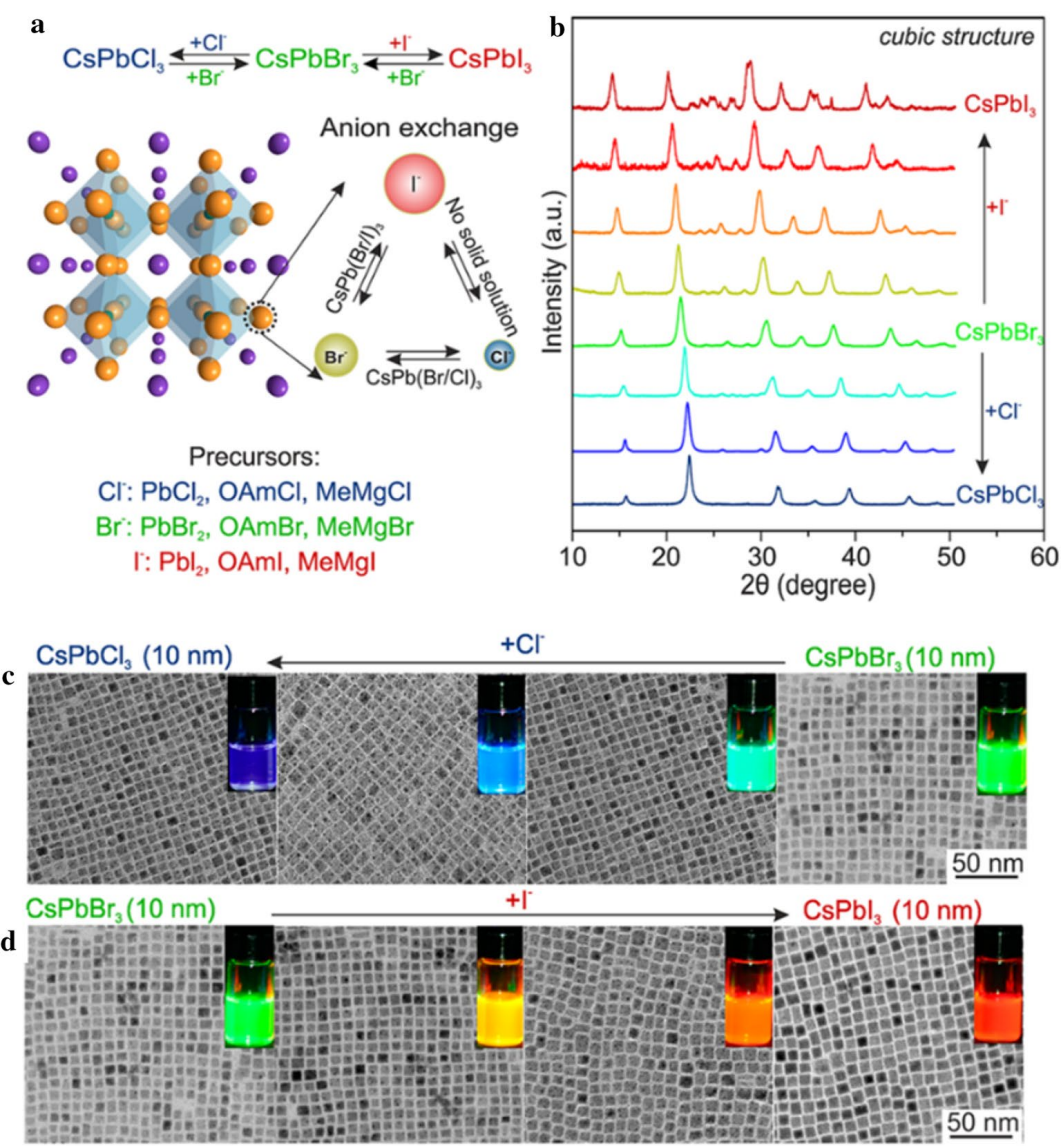
**a** A schematic of the anion exchange within the cubic perovskite crystalline structure of CsPbY_3_. **b** Powder X-ray diffraction patterns of the initial CsPbBr_3_ and anion-exchanged samples. **c**, **d** Transmission electron microscopy images of ~ 10 nm CsPbX_3_ nanocrystals after treatment with various quantities of Cl^−^ and I^−^ anions (Reprinted with permission from American Chemical Society [104])

Anion exchange is a useful method to apply to perovskites for controlling the composition and tuning the bandgap energy. In addition, the photoluminescence property in perovskites can also be controlled by anion exchange. Nedelcu et al. [104] reported quick, low-temperature partial or complete anion exchange that can be controlled in extremely luminescent semiconductor NPs of CsPbY_3_ (Y=Cl, Br, or I). By modifying the halide ratios in colloidal solution, the shining photoluminescence could be adjusted over the whole visible spectral region while a quantum yield of 20–80% was maintained (Fig. 14). Barnabas et al. [106] also demonstrated controlled morphological changes in nanowires and NRs, thereby presenting benefits for actual applications such as optoelectronic devices. The successful low-temperature growth of a perovskite NR array was studied as well as an anion exchange technique to convert the CH_3_NH_3_PbBr_3_ NR array into CH_3_NH_3_PbI_3_ while preserving the perovskite morphology. Akkeman et al. [103] investigated tuning the optical properties of cesium lead halide perovskite NPs by anion exchange reactions. They reported that the chemical composition and optical properties were finely tuned from green-emitting CsPbBr_3_ to bright emitters in other regions of the visible spectrum. Parobek el al. [112] reported excited electrons induced by light transfer from CsPbY_3_ NPs to dihalomethane solvent molecules produced halide ions by reductive dissociation, which was accompanied by anion exchange. Controlling either the photons or wavelength of the excitation light was able to precisely tune the extent of the anion exchange reaction.

**Fig. 15:**
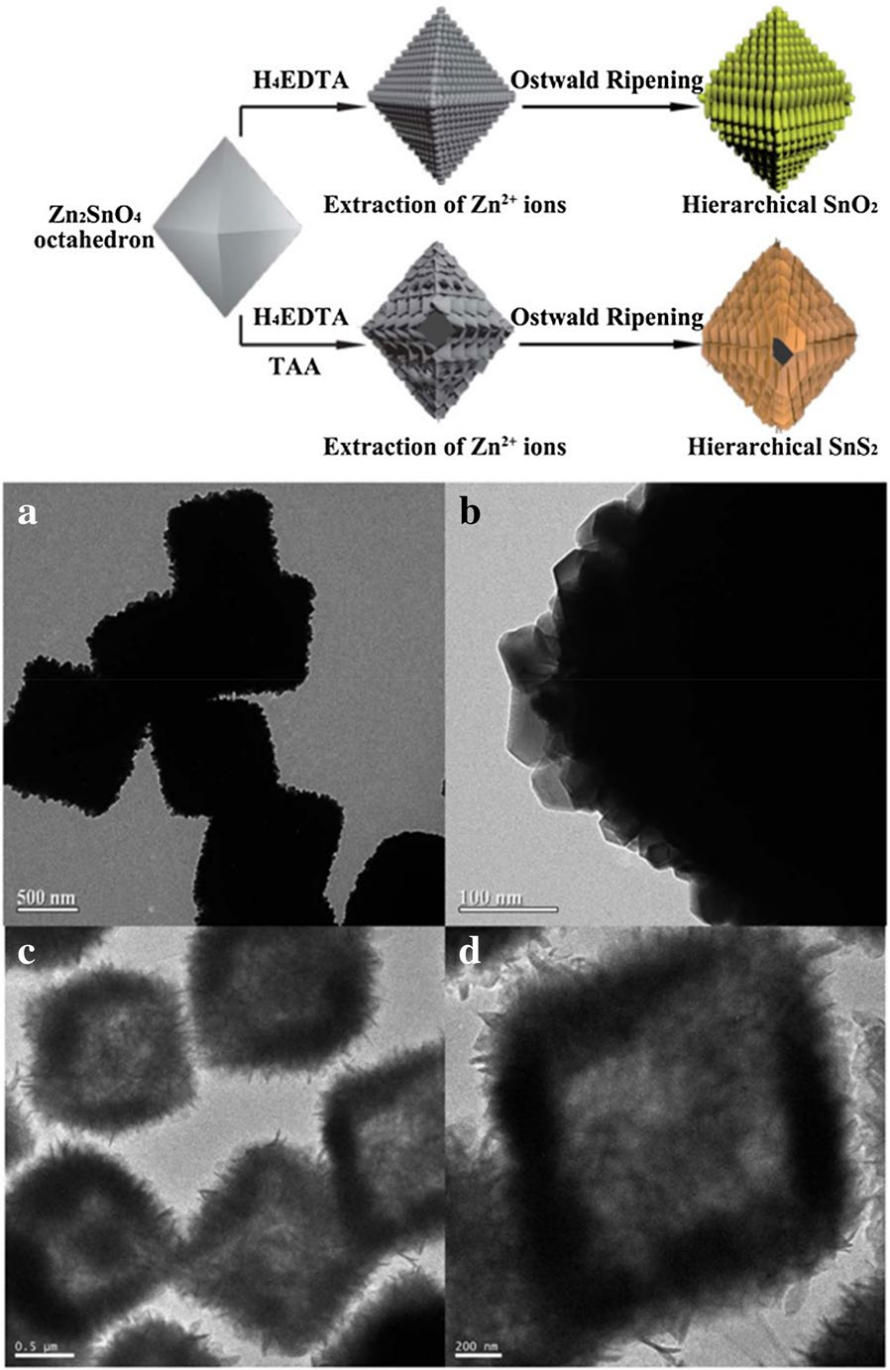
(1) A schematic of the chemical conversion process of Zn_2_SnO_4_ octahedrons to hierarchical SnO_2_ and SnS_2_ octahedrons. (2) Transmission electron microscopy images of SnO_2_ octahedrons at low magnification (**a**) and high magnification (**b**). SnS_2_ octahedrons at low magnification (**c**) and high magnification (**d**) (Reprinted with permission from the Royal Society of Chemistry [113])

In the metal-chalcogenide groups, metal oxides serving as a template have been extensively used for anion exchange due to their high stability compared to other reduced metals which are sensitive to oxidation [98]. In the anion exchange reactions by using the chalcogenide groups, it has been reported that the orientation of metal-oxide NPs is well preserved [98]. In addition, the slow anion diffusion could induce the formation of heterostructured NPs as intermediate products by controlling the reaction kinetics [99, 109]. Dawood et al. [110] observed that wurtzite ZnO NPs can serve as structural templates for the controllable formation of wurtzite ZnS and ZnSe. Zhang et al. [111] demonstrated the anion exchange from CoO to Co_3_S_4_ by using (NH_4_)_2_S, which is a highly reactive precursor even at temperatures as low as 70 °C. Although the Co_x_S_y_ NPs synthesized with (NH_4_)_2_S from initial CoO NPs showed poor crystallinity, this was improved after an annealing process conducted at an elevated temperature (180 °C). The study of anion exchange by using (NH_4_)_2_S has shown that (NH_4_)_2_S can easily supply S^2−^, thus validating that it can be an excellent reagent for altering anions by S^2−^. Cai et al. [113] reported the selective conversion reaction from a ternary Zn_2_SnO_4_ octahedron to SnO_2_ or SnS_2_ by using ethylenediaminetetraacetic acid for extracting Zn^2+^ and thioacetamide as an S precursor (Fig. 15). Xiong et al. [101] investigated the anion exchange of hybrid Cu_2_O/PVP (polyvinylpyrrolidone) NPs by using thiourea. The hybrid NPs containing Cu_2_O crystallites induced the slow diffusion of thiourea to the Cu_2_O NPs surface as well as promoting the diffusion of Cu to the NP surface. These diffusion processes controlled by PVP led to a hybrid core/Cu_2_S shell with multiple layers. Anion exchange has also been investigated in the conversion reaction of nanosheets from Co_3_O_4_ to CoS, NiO to NiS, and WO_3_ to WS_2_ [114, 115].

**Fig. 16:**
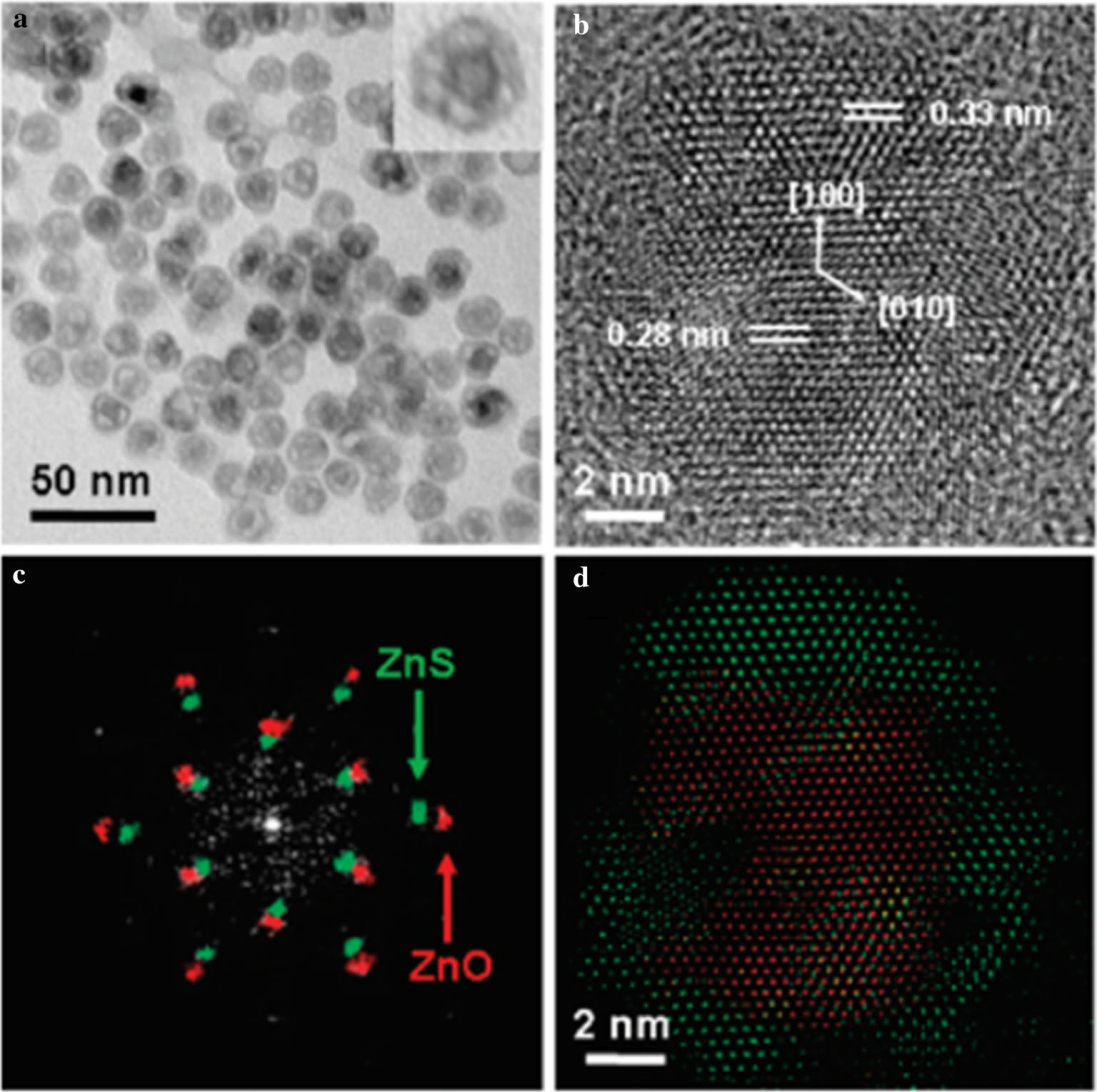
**a** A transmission electron microscopy image of the partially exchanged yolk-shell NPs and **b** a high-resolution transmission electron microscopy image and fast Fourier transform (**c**) of a single yolk-shell NP. **d** The reconstructed crystal structure from (**c**) (Reprinted with permission from American Chemical Society [109])

**Fig. 17:**
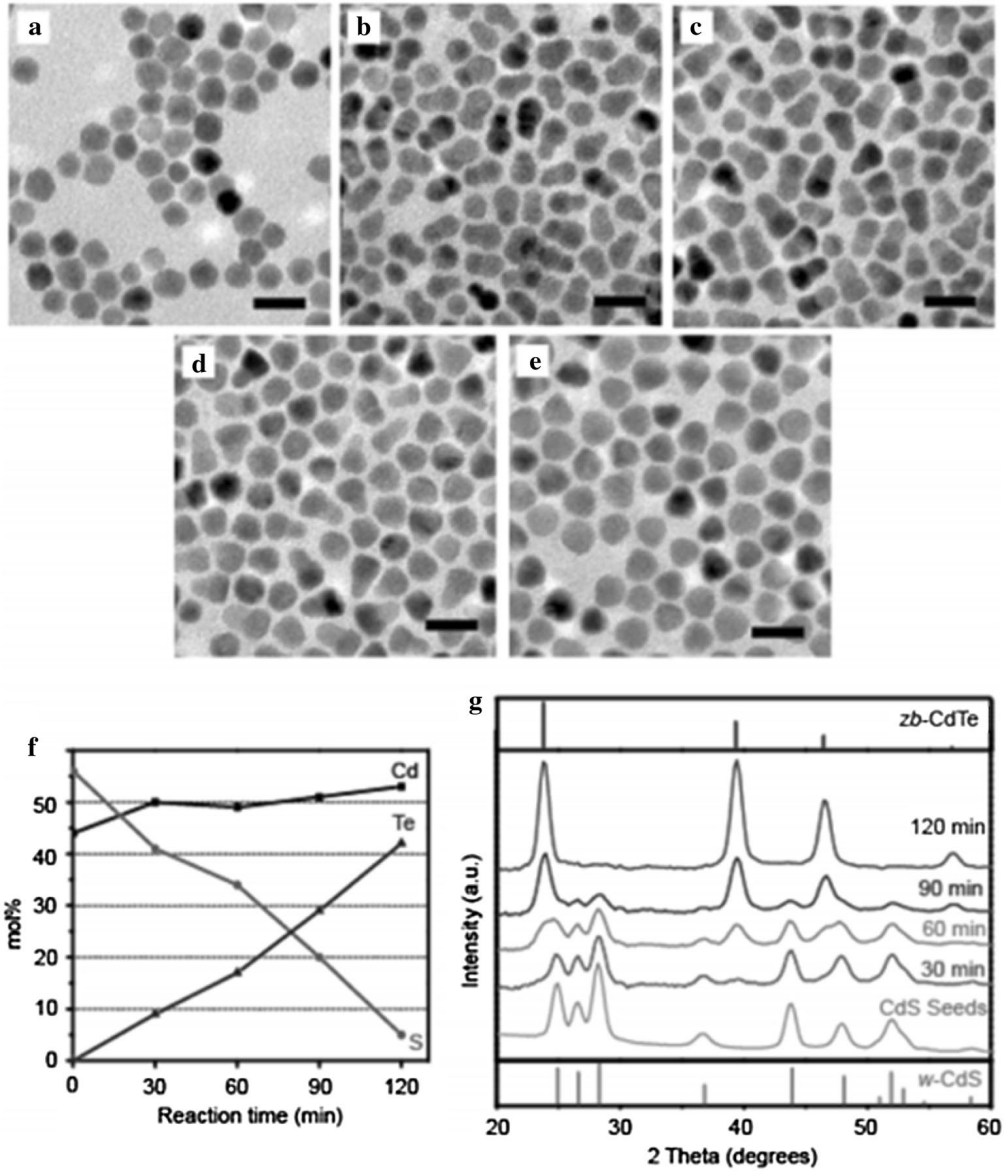
**a**–**e** Transmission electron microscopy images of the products synthesized by the anion exchange reaction of wurtzite CdS NPs with TOP = Te at 260 °C for **a** 0, **b** 30, **c** 60, **d** 90, and **e** 120 min. **f** Temporal change in the composition as estimated by X-ray fluorescence. **g** The crystal structure during the anion exchange reaction of 10 nm CdS NPs at 260 °C (Reprinted with permission from American Chemical Society [99])

Heterostructured NPs can be obtained by anion exchange reactions accompanied by slow reaction kinetics. Park et al. [109] reported the anion exchange from ZnO to solid, hollow structures of monocrystalline ZnS NPs. The morphology and crystalline symmetry of the anion-exchanged ZnS NPs were well preserved along with the atomic orientation of the original ZnO NPs. This study of the anion exchange from ZnO to ZnS showed that the chemical transformation of NPs followed by the ‘Kirkendall effect’ generally results in polycrystalline NP products, while monocrystalline NPs are hardly ever obtained (Fig. 16). Saruyama et al. [99] performed the anion exchange reaction of CdS NPs with TOP telluride (TOP = Te) to produce CdS/CdTe heterodimers; the spontaneous formation of these occurred due to the strain relaxation related to the different crystalline orientations of the wurtzite CdS and zincblende CdTe phase (Fig. 17).

## Conclusions

NP synthesis through ion exchange is noted as one of the most advanced synthetic methods due to its versatility leading to novel NPs with complex heterostructures (e.g. core–shell, segmented, etc.) and thermodynamically metastable phases, which are considered as limitations in conventional synthesis methods. The main factors controlling ion exchange are the thermodynamic and kinetic properties of the ions. As demonstrated by many examples of ion exchange reactions yielding new NPs, this is a promising state-of-the-art technique to explore new nanostructured materials and provide new design principles for nanosynthetic approaches.

## Data Availability

Not applicable.
